# Spatial and Temporal Variation of Archaeal, Bacterial and Fungal Communities in Agricultural Soils

**DOI:** 10.1371/journal.pone.0051554

**Published:** 2012-12-20

**Authors:** Michele C. Pereira e Silva, Armando Cavalcante Franco Dias, Jan Dirk van Elsas, Joana Falcão Salles

**Affiliations:** 1 Department of Microbial Ecology, Centre for Life Sciences, University of Groningen, Groningen, The Netherlands; 2 Department of Soil Science, “Luiz de Queiroz” College of Agriculture, University of São Paulo, Piracicaba, São Paulo, Brazil; U. S. Salinity Lab, United States of America

## Abstract

**Background:**

Soil microbial communities are in constant change at many different temporal and spatial scales. However, the importance of these changes to the turnover of the soil microbial communities has been rarely studied simultaneously in space and time.

**Methodology/Principal Findings:**

In this study, we explored the temporal and spatial responses of soil bacterial, archaeal and fungal β-diversities to abiotic parameters. Taking into account data from a 3-year sampling period, we analyzed the abundances and community structures of *Archaea*, *Bacteria* and *Fungi* along with key soil chemical parameters. We questioned how these abiotic variables influence the turnover of bacterial, archaeal and fungal communities and how they impact the long-term patterns of changes of the aforementioned soil communities. Interestingly, we found that the bacterial and fungal β-diversities are quite stable over time, whereas archaeal diversity showed significantly higher fluctuations. These fluctuations were reflected in temporal turnover caused by soil management through addition of N-fertilizers.

**Conclusions:**

Our study showed that management practices applied to agricultural soils might not significantly affect the bacterial and fungal communities, but cause slow and long-term changes in the abundance and structure of the archaeal community. Moreover, the results suggest that, to different extents, abiotic and biotic factors determine the community assembly of archaeal, bacterial and fungal communities.

## Introduction

Understanding temporal and spatial patterns in the abundance and distribution of communities has been a fundamental quest in ecology. Such an understanding is crucial to allow an anticipation of responses of ecosystems such as soil to global changes [1]. Because local conditions are never constant, small disturbances that affect the soil microbial communities might occur [2]–[3] at different temporal and spatial scales. The assessment of microbial communities at a particular locality may result in patterns that vary greatly both within and between years, and these communities may be subjected to changes over longer time scales as a result of processes such as succession and evolutionary change [4]. One approach to investigate temporal (and spatial) variability in complex systems is to explore patterns of β-diversity. Whereas alpha (α-) diversity represents a measure of the total diversity of a given site, β-diversity is the variation of species composition (turnover) across space or time between paired sites. High β-diversity indicates large differences in community composition among different sites. Such high diversity can result from local as well as regional factors, e.g. changes in the local environmental conditions or limitation of dispersal between sites [5].

Temporal variation of conditions is a very common feature of ecosystems. Ecologists have long been interested in how such variation structures natural communities [6], [7]. It can presumably affect the rate of microbial turnover, as microorganisms can process resources and adapt to changes in natural environments on a much faster time scale than macroorganisms [8]. Moreover, many functional microbial groups can show dramatic seasonal changes in soils [9].

The number of studies employing the concept of β-diversity to understand how microbial communities respond to biotic and abiotic parameters has increased substantially in soil ecology. Martiny and co-workers [10] studied the mechanisms driving ammonia-oxidizing bacterial (AOB) communities in salt marsh sediments. They found no evolutionary diversification when comparing the AOB community composition between three continents; although a negative relationship was observed between geographic distance and community similarity. Furthermore, in an attempt to determine to which extent a bacterial metacommunity that consisted of 17 rock pools was structured by different assembly mechanisms [11], the authors studied changes in β-diversity across different environmental gradients over time, including phosphorus concentration, temperature and salinity. They found that there were temporal differences in how the communities responded to abiotic factors. β-diversity allows not only the understanding of temporal but of spatial variations as well. For instance, in a survey of bacterial communities across more than 1000 soil cores in Great Britain [12], no spatial patterns were observed, but instead variations in β-diversity according to soil pH were found, which revealed that β-diversity (between sample variance in α-diversity) was higher in acidic soils (pH 4–5) than in more alkaline soils (pH 7–9) [12]. In the former soils, environmental heterogeneity was highest, calculated as the variance in environmental conditions [12]. In another study, different patterns of bacterial β-diversity were observed between different layers in sediment cores, which could be attributed to historical variation and geochemical stratification [13].

Of the soil microbial groups, bacteria have been mostly studied, as they exhibit an estimated species diversity of about 10^3^ to up to 10^6^ per g soil [14]–[16]. However, archaea and fungi are also important microorganisms found in soil. Previous studies have shown the ubiquity of archaea in soil, especially the crenarchaeota [17]–[19]. Fungal abundances in the order of 10^4^ fungal propagules per g of dry soil were observed in Antarctic soils [20] and 10^7^ per g of soil in soil crusts [21]. Fundamental differences in the physiology and ecology of members of such communities would suggest that their patterns of spatial and temporal variation are controlled by distinct edaphic factors.

In this study, we explored the temporal and spatial fluctuations of soil microbial communities and their relation to local environmental conditions. In order to do so, we investigated the spatiotemporal dynamics of the soil microbiota by analyzing the patterns of α- and β-diversity of archaea, bacteria and fungi in eight agricultural soils across the Netherlands. We sampled the soils eleven times, from 2009 to 2011. Furthermore, to complement the analyses, we applied TLA (time-lag analysis) [22], a distance-based approach to study the temporal dynamics of communities by measuring community dissimilarity over increasing time lags. TLA provides measures of model fit and statistical significance, allowing the quantification of the strength of temporal community change in a numerical framework [23]. We thus interrogated how the relationship between microbial abundance, species composition and the surrounding environment varies in space and time and how this relates to long-term compositional changes.

## Materials and Methods

### Study area and field sampling

The eight soil sites sampled are located in the Netherlands. Their characteristics and geographical coordinates are found in Table 1 and in Table S1. Sampling points were selected to reflect temporal differences in abiotic parameters. For each soil four replicates were taken. Each replicate consisted of 10 subsamples (15–20 cm deep) collected between plots with a spade, away from the plant roots. Soil samples were collected four times over an annual cycle in 2009 (April, June, September and November), three times in 2010 (April, June and October), and four times in 2011 (February, April, July and September). Each sample was placed in a plastic bag and thoroughly homogenized before analysis. A 100-g subsample was kept at 4°C and used for chemical analyses, whereas the remaining soil was kept at −20°C for subsequent DNA extraction and molecular analysis of community composition and total abundance (see below).

**Table 1:** List of soils included in this study.

Sampling Site	Soil type	Land use	Crops			North coordinate	East coordinate
			2009	2010	2011		
Buinen (B)	Sandy loam	agriculture	barley	potato	potato	52°55′386″	006°49′217″
Valthermond (V)	Sandy loam	agriculture	barley	potato	potato	52°50′535″	006°55′239″
Droevendaal (D)	Sandy loam	agriculture	triticale	barley	barley	51°59′551″	005°39′608″
Wildekamp (K)	Sandy loam	grassland	grass	grass	grass	51°59′771″	005°40′157″
Kollumerwaard (K)	Clayey	agriculture	potato	grass	sugar beet	53°19′507″	006°16′351″
Steenharst (S)	Silt loam	agriculture	grass	potato	grass	53°15′428″	006°10′189″
Grebedijk (G)	Clayey	agriculture	potato	wheat	wheat	51°57′349″	005°38′086″
Lelystad (L)	Clayey	agriculture	potato	grass	corn	52°32′349″	005°33′601″

### Soil chemical analysis

The environmental variables measured included pH, concentrations of nitrate (N-NO_3_^−^ in mg/kg of soil), ammonium (N-NH_4_^+^ in mg/kg of soil), organic matter (OM in %) and clay content (in %). The pH was measured in CaCl_2_ suspension 1∶4.5 (g/v) (Hanna Instruments BV, IJsselstein, The Netherlands). Organic matter (OM) content is calculated after 4 hours at 550°C. Nitrate (N-NO_3_^−^) and ammonium (N-NH_4_^+^) were determined with a colorimetric method using the commercial kits Nanocolor Nitrat50 (detection limit, 0.3 mg N kg-1 dry weight, Macherey-Nagel, Germany) and Ammonium3 (detection limit, 0.04 mg N kg-1 dry weight; Macherey-Nagel, Germany) according to manufacturer's protocol.

### Nucleic acid extraction

DNA was extracted from 0.5 g of soil using Power Soil MoBio kit (Mo Bio Laboratories Inc., NY), according to the manufacturer's instructions, after the addition of glass beads (diameter 0.1 mm; 0.25 g) to the soil slurries. The cells were disrupted by bead beating (mini-bead beater; BioSpec Products, United States) three times for 60 s. Following extraction, the DNA preparations were electrophoresed over agarose gels in order to assess DNA purity, quality (average size) and quantity. The quantity of extracted DNA was estimated on gel by comparison to a 1-kb DNA ladder (Promega, Leiden, Netherlands) and quality was determined based on the degree of DNA shearing (average molecular size) as well as the amounts of coextracted compounds.

### Real-time PCR quantification (qPCR)

Absolute quantification was carried out in four replicates on the ABI Prism 7300 Cycler (Applied Biosystems, Germany). The 16S rRNA gene was amplified by qPCR using diluted extracted DNA as template. Specific primers for archaea (group 1 crenarchaeota) 771F/957R [24] and for V5–V6 region of bacteria 16SFP/16SRP [25] were used. We have chosen to focus on Crenarchaeota, as this group is often more common in soil environments than Euryarchaeota [26]. For Fungal community primers 5,8S/ITS1f [27] were chosen. Cycling programs and primer sequences are detailed in Table S2. The specificity of the amplification products was confirmed by melting-curve analysis and on 1.5% agarose gels. Standard curves were obtained using serial dilutions of plasmid DNA containing the cloned 16S rRNA gene obtained from *Burkholderia terrae* BS001 or ITS region of *Rhizoctonia solani* AG3. Dilutions ranged from 10^7^ to 10^2^ gene copy numbers/µl. The archaeal standard curve was obtained by serial dilution of PCR product generated from *Cenarchaeum symbiosum* with the aforementioned archaeal specific primers [24].

### PCR for DGGE analysis

For DGGE analysis, bacterial 16S rRNA genes were PCR amplified using the forward primer F968 [28] with a GC-clamp attached to 5′ and the universal R1401.1b [29]. Archaeal 16S rRNA genes were amplified with the A2F/U1406R primer pair [30], following amplification using the *Archaea*-specific forward primer at position 344 with a 40-bp GC clamp [31] added to the 5′ end, and a universal reverse primer at position 517. The fungal ITS region was amplified with EF4 [32]/ITS4 [33], followed by a second amplification with primers ITS1f-GC [34]/ITS2 [33]. PCR mixtures, primer sequences and cycling conditions are described in Table S3. About 200 ng of amplicons were loaded onto a 6% (w/v) polyacrylamide gel in the Ingeny Phor-U system (Ingeny International, Goes, The Netherlands), with a 20–50%, 45–65% and 40–60% denaturant gradient for the fungal, bacterial and archaeal community, respectively (100% denaturant corresponded to 7 M urea and 40% (v/v) deionized formamide). Electrophoresis was performed at a constant voltage of 100 V for 16 h at 60°C. The gels were stained for 60 min in 0,5× TAE buffer with SYBR Gold (final concentration 0,5 µg/liter; Invitrogen, Breda, The Netherlands). Images of the gels were obtained with Imagemaster VDS (Amersham Biosciences, Buckinghamshire, United Kingdom). Genetic fingerprints were analyzed using GelCompar software (Applied Maths, Sint-Martens Latem, Belgium) [35], [36].

### Data analyses

The diversity of each of the soil bacterial, archaeal and fungal communities was determined on the basis of the PCR-DGGE profiles. Total diversity (α) of the dominant community members was estimated from these data using the Shannon index, as recommended by Hill et al. [37], as well as the number of DGGE bands (species richness). We calculated the temporal β-diversity of archaeal, bacterial and fungal communities as the mean of all pairwise Bray-Curtis dissimilarities based on the relative abundance of DGGE bands, as previously described [38], [39], [11]. To support results from the calculated β-diversity and to test the statistical significance and strength of community dynamics we used time-lag analysis (TLA) [22] by plotting Hellinger-transformed [40] distance values against the square root of the time lag for all lags. The time-lag analytical approach can produce a number of general theoretical patterns with time-series data [22]. The square root transformation reduces the probability that a smaller number of points at larger time lags will bias the analysis [41]. The Bray-Curtis matrices as well as Hellinger-transformed distances were determined in PRIMER-E (version 6, PRIMER-E Ltd, Plymouth, UK; [42]).

To test how α-diversity, β-diversity and microbial abundance varied in relation to environmental variables, parametric Pearson correlation coefficients were calculated between α and β diversities, soil pH, organic matter, nitrate, ammonium, clay content and soil moisture, as well as between total abundances and TLA slopes using SPSS v18.0.3 (SPSS Inc., Chicago, IL, USA). All variables except pH were transformed (Log(x+1)) prior to all analyses. Moreover, we applied variance partitioning to evaluate the relative contribution of the drivers of the microbial assemblages. Forward selection was used on CCA (Canonical Correspondence Analysis) to select a combination of environmental variables that explained most of the variation observed in the species matrices. For that, a series of constrained CCA permutations was performed in Canoco (version 4.0 for Windows, PRI Wageningen, The Netherlands) to determine which variables best explained the assemblage variation, using automatic forward selection and Monte Carlo permutation tests (permutations = 999). The length of the corresponding arrows indicated the relative importance of the chemical factor explaining variation in the microbial communities.

## Results

### Variability of environmental parameters

Soil pH, nitrate, ammonium and organic matter levels were determined in triplicate across all soil samples. Soil pH was significantly higher (P<0.05) in soils K, G and L (7.32±0.06, n = 57) than in soils B, V, D, W and S (4.88±0.04, n = 99) during the whole experimental period and no significant variation over time was observed. In all soils, significant changes were observed in the levels of nitrate, with lower values at the end of the growing season for most of the soils (September 2009: 32.78 mg/kg±7.77; October 2010: 24.15 mg/kg±3.62; September 2011: 2.45 mg/kg±0.41) and higher at the beginning (April 2009: 75.6 mg/kg±12.5; April 2010: 56.4 mg/kg±5.63; April 2011∶100.1 mg/kg±16.5). Levels of ammonium also varied over the whole period, with higher values being observed at the beginning of the season (April 2009: 13.3 mg/kg±1.14; April 2010: 16.0 mg/kg±1.19; April 2011: 12.1 mg/kg±2.72) and lower values at the end (September 2009: 1.93 mg/kg±0.16; October 2010: 8.86 mg/kg±1.22) (Table S1).

Considering each soil individually, they had characteristically different values, with higher levels of nitrate and ammonium found in soils B, V, D and S than in soils W, K, G and L (Table S1). In 2009 and 2010, variations in organic matter (OM) content were observed from September (5.63%±1.20) to November (7.34%±1.45) 2009 and from April (6.28%±0.85) to June (5.04%±0.89) 2010. Small but insignificant variations in OM were observed in 2011. On average, the OM content of all soils was in the range around 4%, except for soil V, which had on average 17% OM.

### Temporal variations in the abundance of archaeal, bacterial and fungal communities and their responses to abiotic variables

**Figure 1:**
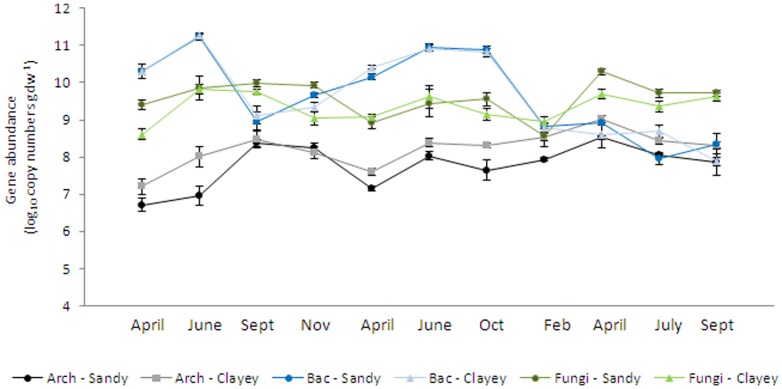
Changes in abundance of archaeal, bacterial and fungal communities. The copy number in each gram of dry soil was estimated by real-time PCR in the eight agricultural soils as an average of sandy and clayey soils at different sampling times. Bars are standard errors (n = 4).

We studied the variations in the abundances of archaeal, bacterial and fungal communities over time and across all samples in three years. The total bacterial abundance showed significant temporal variation during the whole period, ranging between 8.12±0.23 (mean ± standard error) (September 2011) and 10.93±0.06 (June 2010) log copy numbers per g dry soil and showing comparable copy numbers in sandy (9.65±0.13) and clayey soils (9.64±0.16). The archaeal abundance (crenarchaeota) ranged between 6.96±0.14 (April 2009) and 8.78±0.07 (April 2011) log copy numbers per g dry soil, and showed significant differences between sandy and clayey soils across almost all sampling times, with lower numbers in the sandy soils (7.77±0.13) than in the clayey soils (8.22±0.13). Fungal abundance varied between 8.76±0.16 (February 2011) and 10.00±0.09 (April 2011), and significantly higher abundance was observed in the sandy soils depending on the sampling time (Fig. 1). Overall and on average, the abundance of bacteria was higher than that of the fungi, except in September 2009 and during 2011.

We used Pearson's correlation to examine how soil parameters influenced the abundances of bacterial, archaeal and fungal communities. Whereas the archaeal abundances were positively correlated with soil pH (r = +0.883, P<0.001), they were negatively influenced by nitrate (r = −0.764, P<0.05). A positive relationship was observed between fungal abundance and soil organic matter (r = +0.722, P<0.05), and a negative relationship was observed between fungal abundance and archaeal abundance (r = −0.484, P<0.05). Relationships between the abundance of bacteria and fungi, as well as between bacteria and archaea, were not significant. Interestingly, none of the soil parameters measured influenced bacterial abundance significantly.

### Patterns of α-diversity and response to abiotic variables

**Figure 2:**
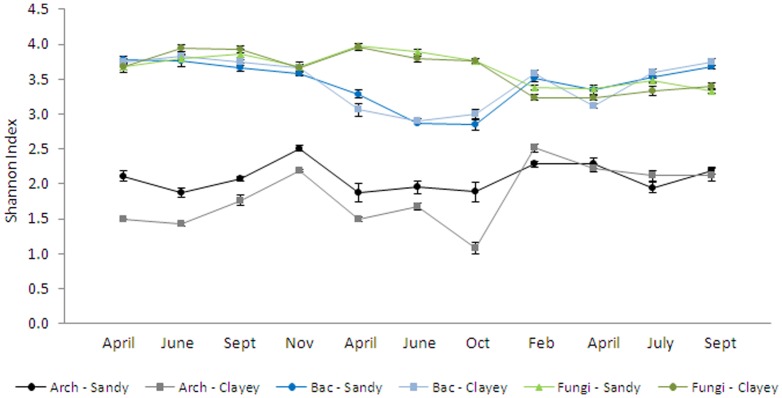
Total α-diversity of archaeal, bacterial and fungal communities. Alpha diversity was calculated as the average of the Shannon Index (H′) per soil type (sandy×clayey), from April 2009 to September 2011 (mean ± s.d.).

Understanding how species are distributed in space and time may yield a first avenue towards their assembly rules [43]. We used two ecological measures, i.e. the Shannon index (H′) and species richness, as proxies to study the variations in the α-diversities of the archaeal, bacterial and fungal communities. Differential patterns of archaeal, bacterial and fungal α-diversities were observed, as measured by H′ (Fig. 2). The H′ values of the archaeal communities ranged from 1.68±0.04 in June 2009 to 2.40±0.05 in February 2011, and they were consistently lower than the corresponding bacterial and fungal values. The bacterial H′ values varied from 2.52±0.04 in October 2010 to 3.85±0.04 in April 2009, whereas those of the fungal communities varied from 3.2±0.16 in April 2009 to 4.09±0.04 in April 2010 (Fig. 2). In general, the differences observed between sandy and clayey soils for the bacterial and fungal diversities (Shannon index) were time point-dependent. For archaea, a higher Shannon index was noticed in the sandy soils compared to the clayey ones in 2009 and 2010 but not in 2011.

Concerning correlations with edaphic factors, a positive effect of OM content was observed on the archaeal α-diversity (r = +0.691, P<0.05) (Table 2). When using the number of DGGE bands as a measure of α-diversity (species richness), a significant and strong positive correlation was found between archaeal α-diversity and nitrate levels (r = +0.962 , P<0.001) (Table 2). None of the soil parameters measured correlated significantly with bacterial or fungal α-diversity.

**Table 2:** Pearson's correlation coefficient between soil chemical parameters, biotic parameters (total abundance, alpha diversity, beta diversity and slopes from TLA analysis), calculated from the eight soils over time.

	*pH*	*N-NH_4_^+^ (mg^−1^kg)*	*N-NO_3_^−^ (mg^−1^kg)*	*OM (%)*	*Clay (%)*
***Total abundance***					
Total archaeal community	**0.883** ***	−0.498^ns^	**−0.764** *	0.030^ns^	**−0.795** *
Total bacterial community	−0.636^ns^	0.379^ns^	0.236^ns^	−0.624^ns^	0.417^ns^
Fungi	0.363^ns^	−0.476^ns^	−0.356^ns^	**−0.722** *	0.387^ns^
***Alpha Diversity (Shannon)***					
Total archaeal community	0.284^ns^	0.230^ns^	0.137^ns^	**0.691** *	0.599^ns^
Total bacterial community	−0.174^ns^	−0.033^ns^	0.470^ns^	−0.158^ns^	0.442^ns^
Fungi	0.095^ns^	−0.149^ns^	0.175^ns^	−0.370^ns^	0.241^ns^
***Alpha Diversity (N° bands)***					
Total archaeal community	−0.408^ns^	−0.056^ns^	**0.962** ***	0.482^ns^	0.442^ns^
Total bacterial community	0.441^ns^	−0.355^ns^	−0.485^ns^	−0.497^ns^	−0.335^ns^
Fungi	−0.154^ns^	0.150^ns^	−0.579^ns^	−0.416^ns^	−0.469^ns^
***Temporal Beta diversity***					
Total archaeal community	−0.194^ns^	−0.249^ns^	**0.874** *	0.541^ns^	−0.415^ns^
Total bacterial community	0.028^ns^	−0.313^ns^	−0.123^ns^	−0.502^ns^	0.074^ns^
Fungi	−0.380^ns^	0.167^ns^	−0.456^ns^	−0.232^ns^	−0.035^ns^

Notes: Values in boldface type indicate significant correlations with P values indicated in superscript.

* P<0.05;

** P<0.01;

*** P<0.001.

ns not significant at P<0.05.

### Patterns of temporal β-diversity and responses to abiotic variables

**Figure 3:**
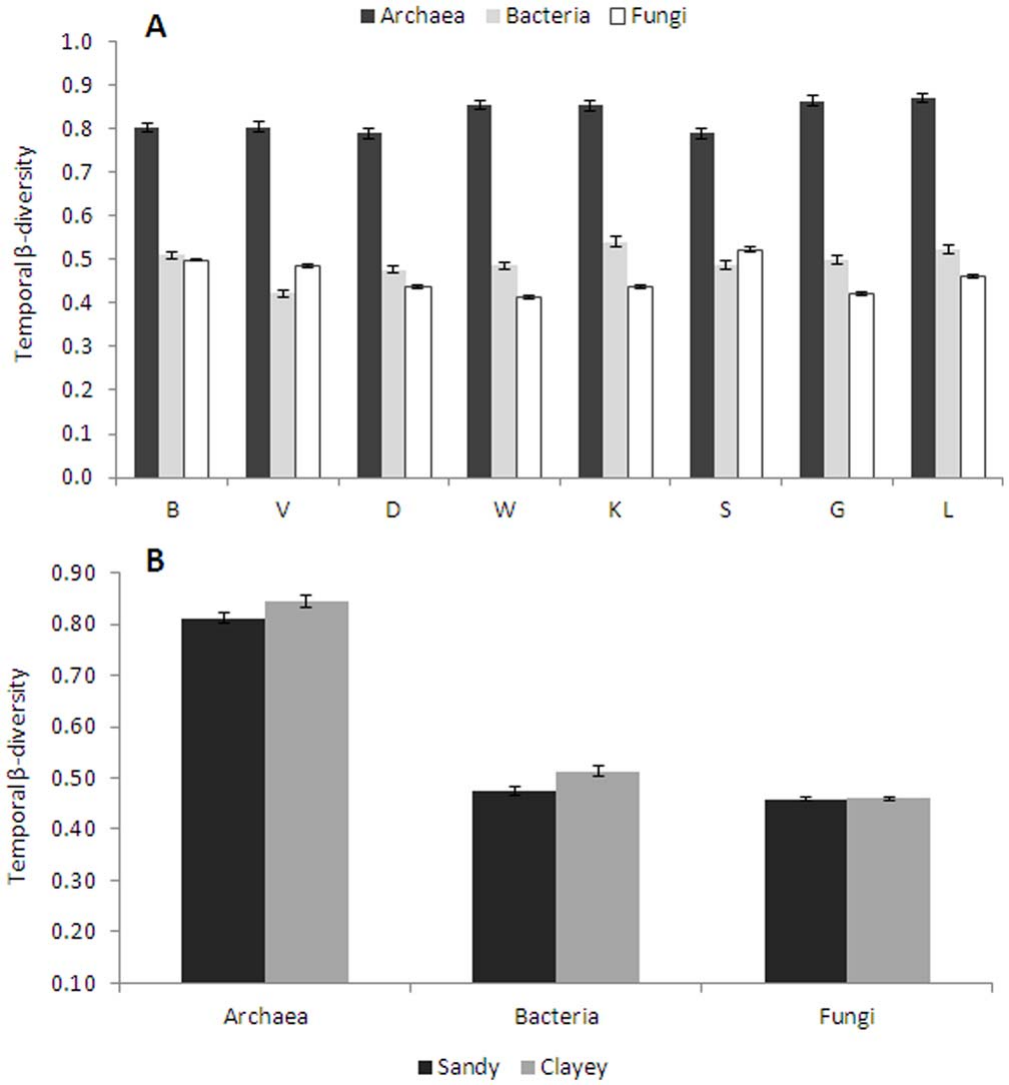
Temporal β-diversity of archaeal, bacterial and fungal communities. Temporal β-diversity, which takes into account temporal changes of each individual soil, was calculated across the different sampling points (A) and separated per soil type (B) (mean ± s.e.).

The patterns of temporal β-diversity of the archaeal, bacterial and fungal communities (taking into account the variations in community composition of each microbial group in individual soils over time) showed small but significant variations across soils (Fig. 3A). Bacterial β-diversities were in general higher than fungal ones across soils, except for soil V. There were slight but significant differences (P<0.05) between sandy and clayey soils regarding the temporal β-diversity of archaeal and bacterial but not of fungal communities (Fig. 3B).

Although chemical parameters might show variability over time, significant correlations could still be observed. The patterns in the archaeal temporal β-diversities observed were mainly due to positive correlations with nitrate (r = +0.874, P<0.05) (Table 2). None of the soil parameters measured were correlated with bacterial and fungal temporal β-diversities. Canonical correspondence analysis was used to test the significance of the influence of soil parameters on the community parameters. We used variance partitioning to control for the effect of each individual parameter, while all others are defined as covariables in the constrained analyses [44]. Considering the whole data set, soil parameters explained 45%, 6.6% and 6.9% of the temporal variability in archaeal, bacterial and fungal community structures, respectively. The archaeal community was mostly affected by changes in OM (11.9%) and nitrogen specimens (nitrate + ammonium; 7.8% each), whereas the bacterial and fungal community variations were mostly related to ammonium (2.1% and 2.2% for bacteria and fungi, respectively) (Fig. S1).

Significant relationships were observed between variation in β-diversity and H′ for bacterial (r = +0.602, P<0.05) and fungal communities (r = −0.481, P<0.05) but not for archaeal communities.

### Quantifying temporal changes of archaeal, bacterial and fungal communities

**Figure 4:**
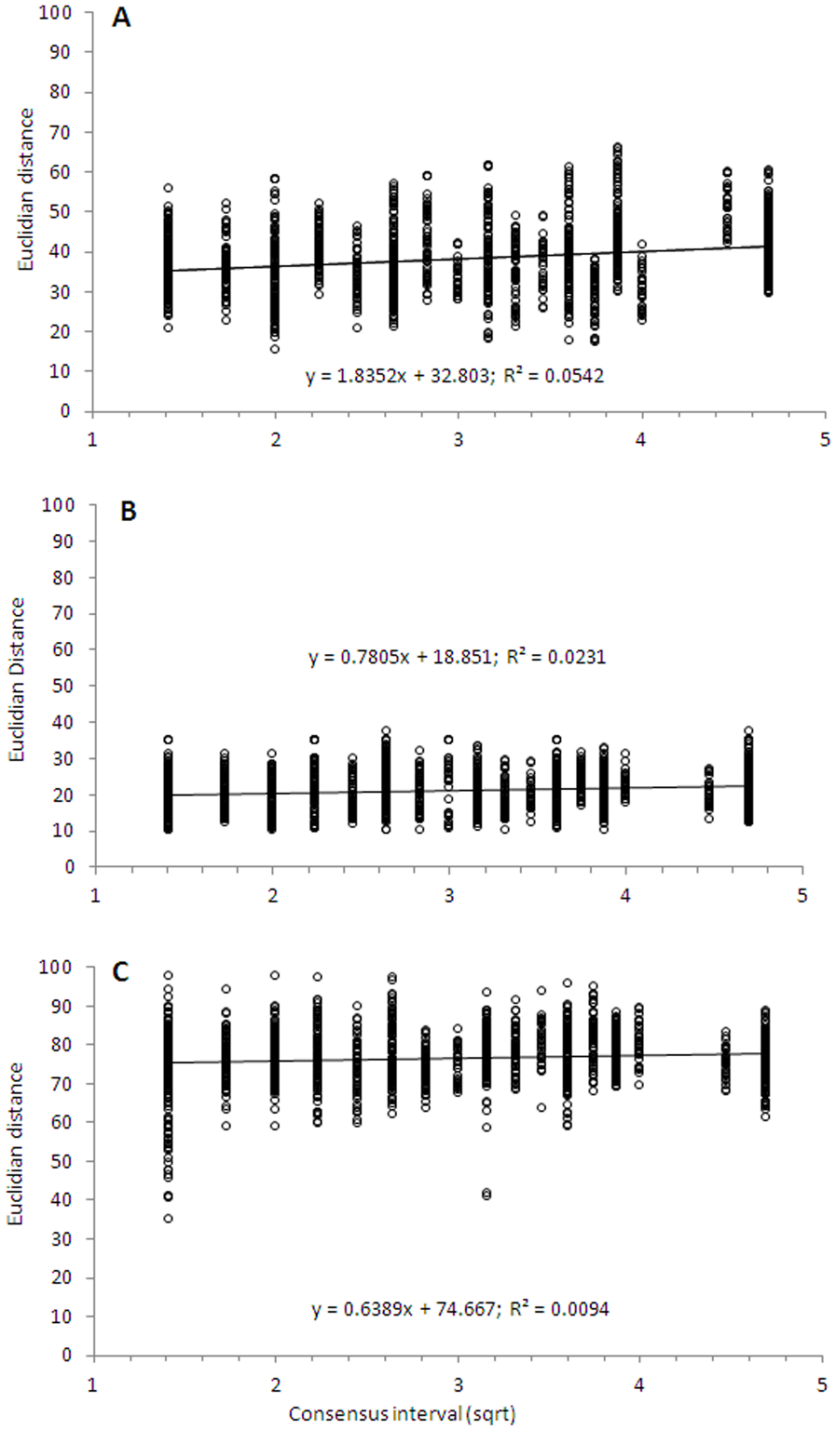
Quantification of archaeal, bacterial and fungal dynamics. Patterns of change (regression of square root of time-lag on Euclidian distance) of archaeal (A, slope 1.835), bacterial (B, slope 0.785) and fungal community (C, slope 0.638) in eight soils. The best-fit line is shown.

The temporal changes of the microbial guilds were quantified and statistically tested via TLA. TLA analyses were performed separately per year and also considering all three years. For both analyses, the results and conclusions were similar. Therefore, we decided to include only the whole three year dataset. A statistically significant regression line (P<0.05) was observed for the archaeal community, with an overall slope of 1.835 (Fig. 4A and Table 3). Moreover, all eight soils showed indications of directional changes in community composition, yielding regression lines that were statistically different from zero (P<0.05) with the exception of the G soil (Table 3). Although the slopes were small (Table 3), they were mainly reflected in the positive Pearson correlations with nitrate levels (r = +0.814, P<0.05). The bacterial communities showed a similar trend as observed for the archaeal ones, with significant regression lines and a slope of 0.785 (Fig. 4B and Table 3). Although analyses of the fungal communities in the eight soils showed that only three soils were undergoing directional changes (B, V an L soils), the overall result based on the simultaneous analysis of all soils yielded statistically significant regression lines (slope of 0.638, P<0.05, Fig. 4C). None of the soil parameters measured had significant effects on the rates of change of bacterial and fungal communities. Significant and contrasting relationships were observed between the TLA slopes and the H′ values of archaeal (r = +0.629, P<0.05) and bacterial (r = −0.523, P<0.05) communities but not of fungal communities.

**Table 3:** Results of the time-lag analyses (TLA) performed for bacterial, archaeal and fungal communities for all soils separately, and an overall result considering all soils.

Sampling Site	Archaeal community			Bacterial community			Fungal community		
	Slope	P	R^2^	Slope	P	R^2^	Slope	P	R^2^
Buinen (B)	1.749	0.000	0.116	3.298	0.000	0.227	0.989	0.007	0.031
Valthermond (V)	1.876	0.000	0.064	1.903	0.000	0.139	1.297	0.001	0.059
Droevendaal (D)	2.063	0.000	0.098	1.864	0.000	0.135	0.671	NS	0.013
Wildekamp (W)	1.765	0.000	0.105	1.059	NS	0.036	0.414	NS	0.004
Kollumerwaard (K)	1.378	0.028	0.021	1.860	0.009	0.079	−0.078	NS	0.000
Steenharst (S)	2.038	0.000	0.080	1.697	0.015	0.069	0.817	NS	0.012
Grebedijk (G)	0.853	NS	0.022	2.300	0.000	0.156	−0.029	NS	0.000
Lelystad (L)	1.282	0.016	0.026	0.552	NS	0.009	0.981	0.011	0.034
*Overall*	1.835	0.000	0.054	0.785	0.000	0.023	0.638	0.000	0.009

## Discussion

### Temporal variation in the abundance of soil microbial communities

In our study, population sizes of archaea, bacteria and fungi, estimated using quantitative PCR, were found to be within the range observed in other soil systems [24], [45]. Quantitative PCR of soil DNA, as any PCR based approach, has its inherent limitations, whether it be the biases of soil DNA extraction, PCR, or the core genes targeted. However, the method is highly reproducible and sensitive, enabling the quantification of microbial abundance changes across temporal and spatial scales. Moreover, this study performed multiple qPCR runs in order to ensure results were statistically significant. In our calculations, we also took into account the efficiency and amount of extracted DNA from the soil samples. Therefore, we argue that our results are representative of the fluctuations observed between different times rather than pure noise.

A high abundance of crenarchaeota in soils has been previously observed [24], [46], possibly indicating a crucial functional role for such organisms in agricultural soils. Furthermore, the bacterial abundance was often higher across soils than the fungal abundance (except at the end of 2009 and the end of 2011), supporting the finding that bacterial∶fungal (B∶F) ratios are quite high in agricultural or grassland soils as compared to, say, forest soils [45], [47]–[49]. These comparisons are very important in the context of whether soils are thought of a being fungal (more “natural”) or bacterial (more highly cultivated) dominated. Indeed, such elevated B∶F ratios may also reflect anthropogenic disturbances due to agricultural practices.

The variations in microbial abundances could be explained by several parameters, depending on the target group. Soil pH and nitrate explained more than 75% of the variation in archaeal abundance. Previous studies have reported negative effects of pH on group 1.1c Crenarchaeota [50] in acid forest soils and negative relationships between nitrate and archaeal abundance [21]. The positive correlations between archaeal abundance and soil pH observed here suggest that our soils might be dominated by crenarchaeal species that are adapted to conditions of higher soil pH (7.0–7.5) [51], which may be linked to the long agricultural history of the plots studied here.

Interestingly, the bacterial abundances didn't respond to soil pH or any other measured abiotic parameter, although several studies have reported pH as the main determinant of bacterial community composition [52]–[57]. It has been shown that some specific bacterial taxa abundances decrease or increase with a changing pH, for instance members of the *Acidobacteria* and *Actinobacteria* [56]. Although pH may have driven changes in the relative abundance of some bacterial classes, the abundance of total bacteria remained quite constant in the different pH ranges, indicating that the carrying capacity of the soil was not strongly affected by pH. Fungal abundance was also not affected by pH, but this was expected since the pH range in our soils was within the (wide) pH optimum for this group, often covering 5–9 pH units without significant inhibition of growth [58], [59]. We also observed that when conditions apparently favored increases in fungal abundance, archaeal abundance decreased, suggesting that fungi and archaea might compete for similar niches. Nonetheless, fungal abundance was positively affected by OM content, which is consistent with the saprophytic status of most fungi [60].

### Temporal variation in α-diversity

None of the soil parameters measured in this study were able to explain the patterns of α-diversity observed for bacteria and fungi. It might be that the taxonomic scale was too broad and a deeper analysis would allow a better understanding of the observed patterns, as observed by Rasche et al. [61]. With PCR-DGGE, only the most abundant taxa, comprising no more than 0.1–1% of the community, can be detected. In other words, only the most abundant organisms are detected with PCR-DGGE. Because of these caveats, the parameters calculated from PCR-DGGE fingerprints and correlations based thereon should be interpreted as indications and not as absolute conclusions.

Archaeal α-diversity, on the other hand, was shown to respond to nitrate and OM levels. Nitrate had opposite effects on archaeal richness and diversity, depicting a community that responds to increasing nitrate with an increase of richness but a great decrease of evenness. This most likely indicates the outgrowth of previously undetectable OTUs. Thus, in addition to the strong negative effect on archaeal abundance observed by qPCR, nitrate availability seems to be a crucial factor determining archaeal community structure. The positive correlation between archaeal diversity and soil OM content indicate that the OM provides a substantial fraction of carbon to the local archaeal communities. Recently, genomic analyses of *Crenarchaeum symbiosum* and *Nitrosopumilus maritimus* suggested that these organisms are capable of mixotrophy [62], [63] and that group 1.1c Crenarchaeota are able to grown on methanol and methane [64]. This suggests that archaea might not be solely sustained by ammonia oxidation [65], [66].

### Variation in β-diversity over time (species turnover)

To assess how dynamic each soil microbial group was over time, we calculated the temporal β-diversity (average Bray Curtis dissimilarity) for each soil over time and for each microbial group. We observed a higher temporal β-diversity for archaea than for bacteria and fungi across all soils. This indicates that the archaeal communities are much more dynamic than the bacterial or fungal ones along a time gradient. These differences are probably due to the differential physiologies and sensitivities to environmental perturbations of these microorganisms. It has been shown that changes in temperature, moisture [61], [67] and resource availability due to seasonal variation [61] can affect soil archaeal as well as bacterial communities. Moreover, a clear pattern was observed for bacterial β-diversity in the metacommunity of 17 rock pools, with higher variations during summer and lower during autumn [11]. The temporal variations of archaeal and bacterial communities were also higher in the clayey soils than in sandy ones, suggesting that the latter harbors more dynamic communities.

One main finding of this study is that, although the β-diversity patterns of the three microbial domains investigated are related with the same set of abiotic factors, the total percentage of variation able to explain those patterns was much higher for archaeal (45.0%) than for bacterial (6.6%) or fungal (6.9%) communities. This suggests that the archaeal communities might be much more sensitive to environmental changes than the bacterial or fungal ones. Based on these results, we hypothesize that the archaeal communities of agricultural soils with a long history of N-fertilization are more sensitive to disturbances than the corresponding bacterial or fungal communities.

### Quantification of β-diversity

To be able to quantify community dynamics, allowing comparisons and providing a general overview of long-term trends in the complex soil system, we used the slopes obtained from TLA. TLA has been intensively used to identify directional changes and to quantify temporal dynamics of macroorganisms [69]–[73]but very few studies have focused on microorganisms. Although the TLA slopes for archaea and bacteria were small, they were significantly different from neutral. Clearly, even small changes can be part of a long-term trend. On the contrary, changes in the fungal communities were non-significant, suggesting stochastic species dynamics.

Changes in environmental variables within soil sites determine how time affects turnover (β-diversity), as different microbial interactions are favored if prevailing conditions change [74]. Only archaeal communities responded to changes in environmental parameters, being strongly correlated with nitrogen availability and with the degree of temporal variation quantified by TLA. This might suggest that at some level strongly deterministic processes are acting on the archaeal but not on the bacterial and fungal communities in these soils. Another explanation is that archaea are much more limited in their ecoversatility, whereas bacteria and fungi are highly functionally redundant. The observed relation between bacterial richness and TLA slopes, e.g. high turnover at low richness, was also noticed in a study of the distribution of British birds [75]. The authors discuss that low species richness areas tend to have relatively more random mixtures of species than high species richness areas. The relation observed between archaeal turnover and species richness suggests a less random distribution of species, caused mainly by nitrate contents.

In this study we demonstrate that changes in the community composition of bacteria and fungi could be linked to both environmental and biotic factors (e.g. species-species interactions), as their (α-) diversity co-varied significantly with their β-diversity over the time period of the study. Conversely, archaea showed no significant correlation between α- and β-diversity, and the community shifts were mainly driven by the surrounding environment, mostly by the effects of soil pH and nitrate concentrations. This might indicate that changes in archaeal community are mostly driven by environmental factors, as previously observed by Zinger et al. [68] in a study on the patterns of archaeal, bacterial and fungal communities in an alpine landscape. Furthermore, we propose that different environmental and biological mechanisms act on each microbial niche. A more comprehensive understanding of the rules governing these important soil microorganisms will require additional field work as well as microcosm experiments to identify the key environmental and biotic factors driving the assemblage of these communities.

#### Ethic statement

No specific permits were required for the described field studies. The locations are not protected. The field studies did not involve endangered or protected species.

## Supporting Information

Figure S1 **Biplots of canonical correspondence analysis (CCA) of Archaeal, Bacterial and Fungal similarity matrices and vector fitting of the environmental variables.** Similarity matrices from DGGE data were obtained from eight soils over three years (2009, 2010 and 2011). Physico-chemical data, soil moisture (Humidity), soil nitrate (NO3), soil ammonium (NH4), organic matter (OM), clay content (clay + silt) and soil pH (pH) are presented with black arrows.
(DOCX)

Table S1 **Soil chemical parameters measured in this study.**
(DOCX)

Table S2 **PCR mixtures for real time quantification of Archaeal 16S rDNA, Bacterial 16S rDNA and Fungal ITS region.**
(DOCX)

Table S3 **PCR mixtures for DGGE analysis of Archaeal 16S rDNA, Bacterial 16S rDNA and Fungal ITS region.**
(DOCX)
