# Supplementary material for: Spatial and Temporal Variation of Archaeal, Bacterial and Fungal Communities in Agricultural Soils
Source: PLoS One. 2012 Dec 20;7(12):e51554. doi: 10.1371/journal.pone.0051554 (PMC3527478; doi:10.1371/journal.pone.0051554)
Supplement: Figure S1 — Biplots of canonical correspondence analysis (CCA) of Archaeal, Bacterial and Fungal similarity matrices and vector fitting of the environmental variables. Similarity matrices from DGGE data were obtained from eight soils over three years (2009, 2010 and 2011). Physico-chemical data, soil moisture (Humidity), soil nitrate (NO3), soil ammonium (NH4), organic matter (OM), clay content (clay + silt) and soil pH (pH) are presented with black arrows. (DOCX) [file pone.0051554.s001.docx]

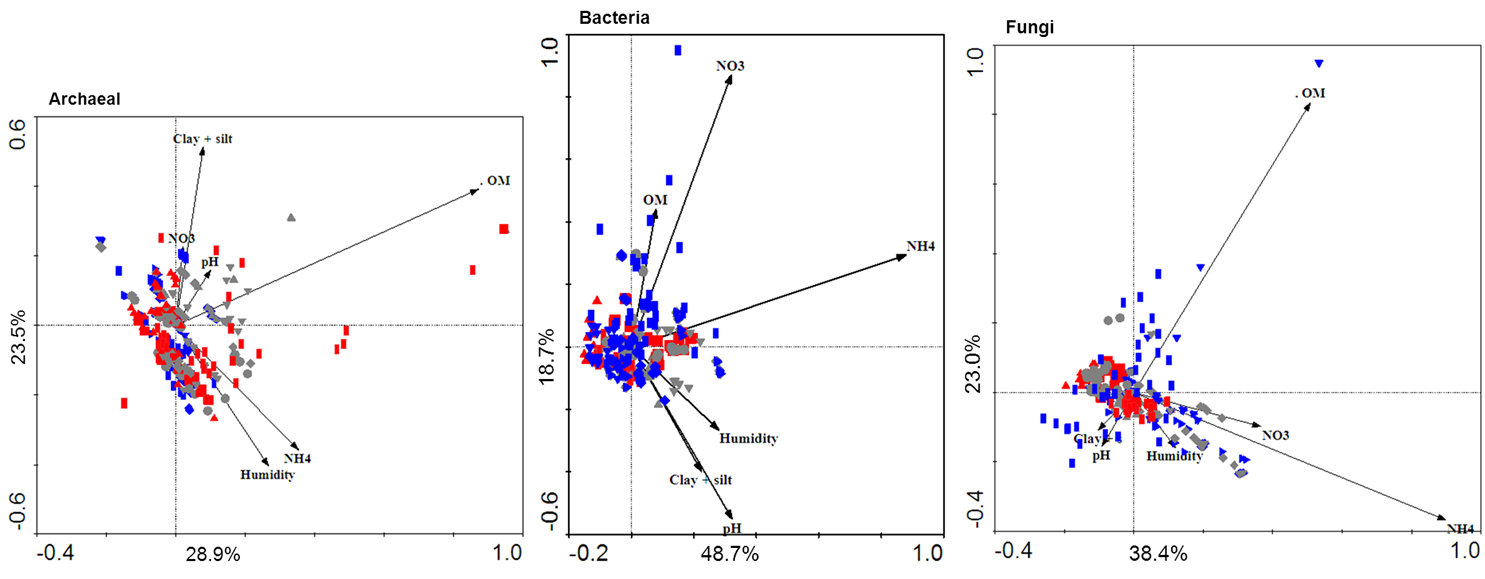


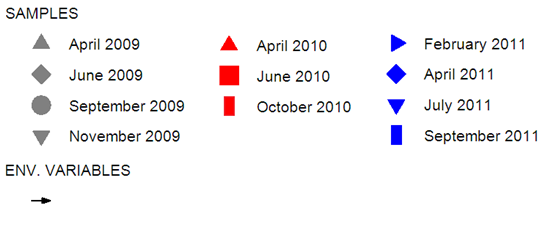


Figure S1: Biplots of canonical correspondence analysis (CCA) of Archaeal, Bacterial and Fungal similarity matrices and vector fitting of the environmental variables. Similarity matrices from DGGE data were obtained from the eight soils over three years (2009, 2010 and 2011). Physico-chemical data, soil moisture (Humidity), soil nitrate (NO3), soil ammonium (NH4), organic matter (OM), clay content (clay + silt) and soil pH (pH) are presented with black arrows.
