# Supplementary material for: Spatial and Temporal Variation of Archaeal, Bacterial and Fungal Communities in Agricultural Soils
Source: PLoS One. 2012 Dec 20;7(12):e51554. doi: 10.1371/journal.pone.0051554 (PMC3527478; doi:10.1371/journal.pone.0051554)
Supplement: Table S1 — Soil chemical parameters measured in this study. (DOCX) [file pone.0051554.s002.docx]

Table S1: Soil chemical parameters measured in this study.
