# Supplementary material for: Spatial and Temporal Variation of Archaeal, Bacterial and Fungal Communities in Agricultural Soils
Source: PLoS One. 2012 Dec 20;7(12):e51554. doi: 10.1371/journal.pone.0051554 (PMC3527478; doi:10.1371/journal.pone.0051554)
Supplement: Table S2 — PCR mixtures for real time quantification of Archaeal 16S rDNA, Bacterial 16S rDNA and Fungal ITS region. (DOCX) [file pone.0051554.s003.docx]

Table S2: PCR mixtures for real time quantification of Archaeal 16S rDNA, Bacterial 16S rDNA and Fungal ITS region

| Real time PCR primers (5’- 3’) | PCR mixtures | Thermal conditions |
| --- | --- | --- |
| ***Archaeal 16S***  771F (ACGGTGAGGGATGAAAGCT)  (Ochsenreiter *et al*., 2003)  957R (CGGCGTTGACTCCAATTG)  (Ochsenreiter *et al.,* 2003) | 12.5µl Power Sybr Green PCR Master mix, 0.5ul BSA (20mg/ml), 0.8µM each primer and 2ul DNA template | 95°C 10 min, 1 cycle  95°C for 30 s, 54°C for 30 s,  72ºC for 30 s, 39 cycles |
| ***Bacterial 16S***  16SFP (GGTAGTCYAYGCMSTAAACG)  (Bach *et al*., 2002***)***  16SRP (GACARCCATGCASCACCTG)  (Bach *et al*., 2002) | 12.5µl Power Sybr Green PCR Master mix, 0.5ul BSA (20mg/ml), 0.8µM each primer and 2ul DNA template | 95°C 10 min, 1 cycle  95°C for 27s, 62°C for 1 min, 72°C for 30s, 39 cycle |
| ***Fungi***  5,8S (CGCTGCGTTCTTCATCG)  (Vigalys *et al*., 1990)  ITS1f (TCCGTAGGTGAACCTGCGG)  (Gardes and Bruns, 1993) | 12.5µl Power Sybr Green PCR Master mix, 0.5ul BSA (20mg/ml), 0.8µM each primer and 2ul DNA template | 95°C 10 min, 1 cycle  95°C for 1 min, 53°C for 30s, 72°C for 1 min, 40 cycles |
