# Supplementary material for: Spatial and Temporal Variation of Archaeal, Bacterial and Fungal Communities in Agricultural Soils
Source: PLoS One. 2012 Dec 20;7(12):e51554. doi: 10.1371/journal.pone.0051554 (PMC3527478; doi:10.1371/journal.pone.0051554)
Supplement: Table S3 — PCR mixtures for DGGE analysis of Archaeal 16S rDNA, Bacterial 16S rDNA and Fungal ITS region. (DOCX) [file pone.0051554.s004.docx]

Table S3: PCR mixtures for DGGE analysis of Archaeal 16S rDNA, Bacterial 16S rDNA and Fungal ITS region

| PCR-DGGE primers (5’- 3’) | PCR mixtures | | Thermal conditions |  |
| --- | --- | --- | --- | --- |
| ***Archaeal 16S* rDNA**  A2F - TTCCGGTTGATCCYGCCGGA  (DeLong, 1992)  U1406F - ACGGGCGGTGTGTRC  (Koga *et al*., 1993) | 0.2mM dNTPs, 1x buffer (Roche), 0.25µl BSA , 0.5µl DMSO, 0.5µM each primer, 0.2U Taq polymerase (Roche) | 95ºC 5 min  94ºC 1 min, 57.5ºC 30 s, 72ºC 4 min, 35 cycles  final extension of 72ºC 7 min | | |
| ARC344 (*ACGGGGCGCAGCAG GCGCGA)  (Bano *et al*., 2004)  517r (ATTACCGCGGCTGCTGG)  (Bano *et al*., 2004) | 0.2mM dNTPs, 1x buffer (Roche), 0.4µM each primer, 0.5U Taq polymerase (Roche) | 94ºC 5min  94ºC 45s, 65ºC-62ºC 45s 7 cycles  72ºC 30s  94ºC 45s, 62ºC-55ºC 45s 6 cycles  72ºC 30s  94ºC 45s, 55ºC 45s 30 cycles  72ºC 30s  72ºC 10 min | | |
| ***Bacterial 16S***  F968 (*AACGCGAAGAACCTTAC)  (Gomes *et al*., 2001)  R1401.1b (CGGTGTGTACAAGAC CCGGGAACG)  (Brons *and van Elsas*, 2008) | 0.2mM dNTPs, 3.75mM MgCl_2_, 1x buffer (Bioline), 1% formamide, 0.2µM each primer, 2.5U Taq polymerase (Bioline) | 95ºC 5 min  60ºC 1’( - 1º /cycle, until 55ºC); 72ºC 2 min 10 cycles  94ºC 1 min, 55ºC 1 min, 72ºC 2 min 20 cycles  72ºC 10 min | | |
| ***ITS region***  EF4 (GGAAGGGRTGTATTTATTAG)  (Smit *et al*., 1999)  ITS4 (TCCTCCGCTTATTGATATGC)  (White *et al*., 1990)  ITS1f (*CTTGGTCATTTAGAGGA AGTA)  (Gardes and Bruns, 1993)  ITS-2 (GCTGCGTTCTTCATCGAT GC)  (White *et al*., 1990) | 0.2 dNTPs, 2.0 mM MgCl_2_, 1x buffer (Bioline), 0.025µl T4 gene protein, 0.4µM each primer, 2.5U Taq polymerase (Bioline)  0.25mM dNTPs, 2.0mM MgCl_2_, 1x buffer (Bioline), 0.4µM each primer, 2.5U Taq polymerase (Bioline) | 94ºC 5min  94ºC 30s, 55ºC 30s, 72ºC 1 min 30s, 34 cycles  72ºC 5 min  94º C 5min  94º C 30s, 55º C 30s, 72ºC 30s, 34 cycles  72º C 5 min | | |

* Means that a GC-clamp is present (Muyzer *et al*., 2001)
